# Perceptions of Finns with chronic diseases about factors affecting their eHealth literacy: A qualitative interview study

**DOI:** 10.1177/20552076231216395

**Published:** 2023-11-27

**Authors:** Alisa Tamminen, Lotta Virtanen, Timo Clemens, Janna Nadav, Petra Saukkonen, Emma Kainiemi, Tarja Heponiemi, Anu-Marja Kaihlanen

**Affiliations:** 1 611153 Maastricht University, Faculty of Health, Medicine, and Life Sciences, Maastricht, The Netherlands; 2 Department of International Health. CAPHRI – Care and Public Health Research Institute, Faculty of Health, Medicine and Life Sciences, 611153 Maastricht University, Maastricht, The Netherlands; 3 3837 Department of Public Health and Welfare, Finnish Institute for Health and Welfare, Helsinki, Finland

**Keywords:** eHealth, eHealth literacy, chronic disease, healthcare services, digitalisation

## Abstract

**Objective:**

This study aims to describe the factors related to the individual, the system and their interaction, which can affect eHealth literacy from the perspective of people living with one or multiple chronic diseases. As digital solutions are increasingly used in healthcare, perspectives of patients with chronic diseases must be considered.

**Methods:**

The study design was a qualitative, descriptive interview study, gathering the insights of people living in Finland with chronic disease. The individual semi-structured interviews (*n* = 17) were conducted via telephone. The eHealth Literacy Framework was used in the data analysis, with a deductive–inductive approach.

**Results:**

The range of skills included in the eHealth Literacy Framework was widely applied by participants who described themselves as adept at using eHealth environments to manage health-related needs. The participants evaluated online information and took an active role in self-management of their chronic disease. Most importantly, even participants possessing many of the skills in the eHealth Literacy Framework experienced difficulties in using eHealth environments, and the accessibility of eHealth environments was highlighted.

**Conclusions:**

eHealth environments could be useful for health promotion and self-management for people with chronic diseases, but only presuming the environments are adapted to their level of eHealth literacy skills.

## Introduction

Due to the changed patterns of health behaviour and ageing of the population, the number of chronic diseases is increasing,^1^ which burdens healthcare systems.^2,3^ Chronic disease can be defined as a ‘disease or condition…that has been or is likely to be present for at least 6 months, including but not limited to asthma, cancer, cardiovascular illness, diabetes mellitus, mental health conditions, arthritis and musculoskeletal conditions’.^4^ Digitalisation is seen as a potential solution to ease the burden of healthcare.^5–7^ Especially in the light of the recent COVID-19 pandemic, the provision and adoption of digital solutions in all areas, including healthcare services and health information provision, have been accelerated out of necessity.^8–13^ Here, the term ‘eHealth environments’ is used to cover both the health information and the healthcare services provided digitally.

eHealth environments have the potential to benefit people with chronic diseases.^14,15^ For example, previous research has shown that access to electronic medical records offered through patient portals can help them feel more involved in managing their own care.^14^ Electronic medical records combined with electronic messaging have also been shown as a feasible alternative to more resource-intensive healthcare visits for those with chronic diseases, without compromising patients’ treatment or health status.^6^

However, eHealth literacy is a prerequisite for using and benefitting from eHealth environments to maintain and promote health.^16,17^ eHealth literacy is defined as one's ‘ability to seek, find, understand, and appraise health information from electronic sources and apply the knowledge gained to addressing or solving a health problem’.^16^ The eHealth Literacy Framework^17^ suggests that eHealth literacy is multidimensionally determined by aspects related to (a) the individual (ability to process information and engage in own health), (b) the system (access to digital services that work and their correspondence to individual needs) and (c) the interaction between them (ability to actively engage with digital services, feeling safe and in control and being motivated to engage with digital services).

Previous studies among chronically ill patients have associated advanced level of eHealth literacy to better access to health information, self-management of care, health-promoting behaviours and lower mortality.^15,18^ However, the evidence on the relationship between specific clinical outcomes and eHealth literacy is somewhat inconsistent.^18^ There is a strong case for more research examining the tenets of eHealth literacy that positively or negatively impact the use of digital services. This interview study aims to describe the aspects related to the individual, the system and their interaction, which can affect eHealth literacy from the perspective of people living with one or multiple chronic diseases. The insights provided in this study could be utilised in the promotion of eHealth literacy for better self-management of chronic diseases.

## Materials and methods

### Study design

This was a qualitative, descriptive interview study. The eHealth literacy framework was applied to data analysis with the aim to characterise the users of and user interactions with eHealth systems.^17^ For the purposes of this study, the expression ‘digital services’ used in the eHealth Literacy Framework domains was replaced by ‘eHealth environments’. The present study covered all types of health information accessible to the general population via the internet, in addition to digital services. This included any health-related information sources, tools and services offered via the internet. Sources of information offered via the internet are, for example, the patient organization internet pages, the Terveyskylä service, health-related blogs or magazines and health encyclopaedias read via the internet such as Duodecim. Tools offered via the internet are, for example, electronic medical records and health and exercise smartphone applications. Health services offered via the internet include, for example, the MyKanta service, remote consultation with a healthcare professional and the electronic renewal of prescriptions.

### Setting

Chronic diseases pose a large burden to people and the healthcare system in Finland, with around half of the adult population reporting having a chronic disease,^19^ which is higher than the EU average for older adults.^20^ Based on the Institute for Health Metrics and Evaluation (IHME) disease burden data, the estimated prevalence of chronic diseases in Finland has been increasing since the 1990s.^21^ In addition, unmet healthcare needs are more common in Finland than in other Nordic countries and are above the EU's average.^19^

The Finnish population's ability to use digitally provided services is notable. More than 90% of Finns already use some digitally provided service via the internet, albeit health services are less commonly used digitally than other services.^22^ In 2020, one-fifth of Finns reported using health services in a digital format.^22^ eHealth is less frequently used by individuals with a lower education level and vulnerable populations such as older adults.^22–24^ Overall, the degree of digitalisation is high in Finland. The national healthcare guidelines recognise the benefits of digital health,^25^ and the level of digitalisation of the health and welfare services is advanced. Over half of Finns reported searching for health information online in 2020, and 64% of the population reported using the MyKanta pages, which is a national health information exchange service, used for health management.^7^

It should be highlighted that, in Finland, the eHealth discussion was already in full swing prior to the COVID-19 pandemic. Ten years ago, there were already scientific articles published on topics such as eHealth transformation drivers^26^ and with eHealth service design being also researched already in 2016.^27^ To understand the effect of the pandemic on the use of information and communication technologies in Finland, the national statistics database was also looked at. In 2018, prior to the COVID-19 pandemic, 89% of Finns had used the internet in the past 3 months. During the pandemic, between 2019 and 2022, the proportion increased from 90% to 93%. Even if the percentage were to decrease in the years after the pandemic to pre-pandemic level, it would still be high. Prior to the pandemic, the proportion of people reporting having looked for information about sickness, nutrition and health in the past 3 months was already over half of the population. Interestingly, this proportion grew from 65% in 2018 to 76% in 2022.^28^

It is noteworthy that different regions in Finland offer different eHealth solutions in terms of eHealth information and services. The Finnish healthcare system does aim for electronic service provision when viable for the patient,^7^ and national policies outline that digital healthcare services should be used whenever feasible.^7^ However, it is known that many Finns feel that they lack digital skills, and almost one-fifth would like to have guidance in the use of eHealth and eWelfare services.^22^

### Participants

For the purpose of this study, people living with chronic diseases were sought out. Altogether, 17 participants were recruited for this study, as previous qualitative studies have found thematic data saturation after approximately 16 interviews.^29^ Participants were considered eligible if they had been living with one or multiple chronic diseases for at least 6 months at the start of the study, were 18 years of age or older and were able to comprehend written and spoken Finnish. All types of chronic diseases were considered, such as diabetes and chronic skin and lung diseases. The interviews were conducted in Finnish language. The researcher had no prior relationship with the participants.

### Data collection

Convenience sampling was used. The invitation to participate was distributed via four major Finnish patient organisations interested in collaboration, out of which all agreed to distribute the invitation to participate to their members. The patient organisations were encouraged to distribute the information letter to their members through any possible physical meetings, or by email, as well as any other strategies they deemed appropriate, including digital means. Additionally, a news article about the study was published on the DigiIN Project consortium website. This article was also shared on social media by multiple of the involved patient organisations.

People who were interested in participating initiated contact with the researchers themselves to join the study by sending an email or calling the contact researcher. Out of those who expressed interest to participate in the study (*n* = 19), the first 17 were included. As per the recent systematic review, data saturation point was expected between 9 and 17 interviews.^30^ Other qualitative interview studies on eHealth have employed groups of around 10–15 participants to gain deeper insights into eHealth solutions.^31,32^ Here, 17 interviews were deemed appropriate to reach data saturation on topics like experiences with eHealth services. We deduced that the data saturation was already reached at 15 interviews. The 17 participants were provided the information letter detailing the type of the study, the areas covered – eHealth environments, user or non-user experiences and perceptions – as well as the purpose of the study.

The researcher AT conducted individual semi-structured interviews with 17 participants. The interviews were conducted via telephone to reach participants widely across Finland and to not to risk the health of the participants due to the on-going COVID-19 pandemic. Interviews were conducted in June of 2022. The interview guide (see Appendix B) covered the domains of the eHealth Literacy Framework. Thus, the participants were asked to freely describe the types of eHealth information sources they had encountered on the internet and describe their user experiences of eHealth environments. This enabled the participants to reflect the experienced benefits and barriers in the context of the types of eHealth environments they had used. Like reported in the results section, many participants brought up not only physician-recommended or otherwise renowned sources but also experiences with being exposed to questionable information and having had to determine its quality. For de-identification reasons, the participants were not asked to disclose their place of residence. During the interview situation, the interviewer took precautions to reduce interview bias. The interviewer maintained a neutral tone, adhered strictly to the interview guide, used standardised questions as well as refrained from making any comments about the answers given and took notes throughout the interviews.

A pilot interview with a chronically ill volunteer interviewee was conducted, after which the language was adjusted to be simpler. The participants were given the opportunity to familiarise themselves with the definitions of eHealth literacy and eHealth environments in advance. The duration of the interviews was 30–60 minutes per interview. Nine of the conducted interviews lasted approximately 30 minutes, six interviews about 45 minutes and one interview 60 minutes. The interview recordings were transcribed by a trusted partner organisation. In total, 159 pages of transcripts were generated with Times New Roman 12 font and 1.0 spacing.

### Data analysis

A combination of deductive and inductive approach was chosen for content analysis, similarly to the design used by Sandström et al.^33^The domains of the eHealth Literacy Framework^17^ served as the theoretical framework on which the deductive part of the coding of the gathered data was based. With eHealth literacy being an emerging field, only few instruments have been constructed for measuring it.^34^ As digital services are quickly advancing, it was deemed reasonable to stay open not to miss factors that seemed relevant, which may not have been considered when the framework was constructed*.* Thus, an inductive coding approach was used.

The analysis was done using the ATLAS.ti 22 software. With regard to data saturation, the recurrence of ideas started to become evident after the first 10 interviews were conducted. After this, the data saturation increased gradually, with very few new ideas or concepts occurring in the last interviews conducted.

The researcher AT first familiarised themself with the transcripts by reading them over and conducted three rounds of coding for the data. Initial code groups were created in ATLAS.ti for the seven different domains of the eHealth Literacy Framework.^17^ Participants’ ideas and thoughts which corresponded with an eHealth Literacy Framework domain were formed into codes. Each code was a reduction of the original expression of one to three sentences, and the code name was formed to correspond with the original expression as precisely as possible. Each code was moved to a code group, representing one of the domains of the seven eHealth Literacy Framework.^17^ This was done using the code group function in ATLAS.ti. After this, the transcripts were reviewed again.

Ideas which did not clearly fit under a framework domain but related to the study topic were made into codes. A few additional code groups were created based on these codes. Then, all the codes were extracted into Excel and arranged by code group. The coding was reviewed and agreed by a team of researchers. The additional code groups were discussed and in the end also incorporated under the eHealth Literacy Framework domains. Out of these code groups, each represents one of the seven domains of the eHealth Literacy Framework, and sub-categories were made to classify the types of ideas belonging to them. As the sub-categories created per code group were abundant (*n* = 6, *n* = 7, *n* = 12, *n* = 17, *n* = 8, *n* = 9), they were condensed into fewer main categories, so two to three main categories per eHealth Literacy Framework domain (see Appendix A).

Active discussions over data analysis process were prioritised to increase the reliability and minimise the influence of individual researcher's own interpretations and preconceived notions, which could be described as applying the idea of ‘Critical friends’ as described by Smith and Sparkes^35^ in their extensive works regarding qualitative research in health.^35,36^ For this study, some key concepts presented by Smith and Sparkes, specifically ‘Worthiness’, ‘Substantive contribution’ and ‘Focus’, apply. For this project, worth and substantive contribution were provided, with little research having been conducted recently to this depth with the target population of those living with a chronic illness, in the digitally advanced setting on Finland. The research project maintained its unique focus throughout from proposal to the final article. Regarding the ideas about fidelity in qualitative research in health,^35^ the necessary width and groundedness were established. However, in terms of adequate data, not all types of people from the target population of Finns living with a chronic illness were reached to generate data with their insights. Additionally, it would be fruitful in the future to generate more co-created research on this topic. For this study, the idea of generating knowledge which is historically and culturally situated is very relevant, and the value of such knowledge should not be disregarded due to lacking statistical–probabilistic generalisability.^35^

### Ethical aspects

The study obtained ethical approval by the ethics committee at the Finnish Institute for Health and Welfare (THL) (THL/1168/6.02.01/2022). Participants received information about their rights in research, study aims, ethical processing and preservation of data and reporting of results. Participation was voluntary, and each participant provided written informed consent by email prior to data collection. The researcher had some personal understanding about living with chronic disease, which improved their interviewer sensitivity but could have induced some bias.

## Results

### Participants’ characteristics

The average age of the participants (*n* = 17) was 57.7 years (*SD* = 13.23), ranging from 31 to 74. The sample, however, was skewed as the majority (82%) of the interviewees were women, and a relatively many (47%) of the participants had a higher education degree. Among the participants, a few had experience with IT or digital healthcare services due to their current or pre-retirement profession. Therefore, they had more extensive IT skills or knowledge about the eHealth platforms available.

Most of the participants shared information of their chronic diseases, and all sharing and participation were voluntary. Almost half (47%) of the participants mentioned having diabetes, asthma, psoriasis and rheumatism were also mentioned. Two participants were living with a rare disease. One-third (35%) described having multiple chronic diseases. However, the sample also appeared to be skewed towards those who actively used different eHealth environments to manage health-related needs. The participants, for example, used the national MyKanta pages for online prescription renewal and checking their health records. The participants also utilised other available local patient portals from their healthcare providers to, for example, book or change healthcare appointments online. Over 76% of the participants described that they have specific eHealth services available to them in the region they live. The participants were living in eight different provinces of Finland. Thus, insights were gained from many regions in Finland, which provided different types of eHealth services.

**Figure 1.**
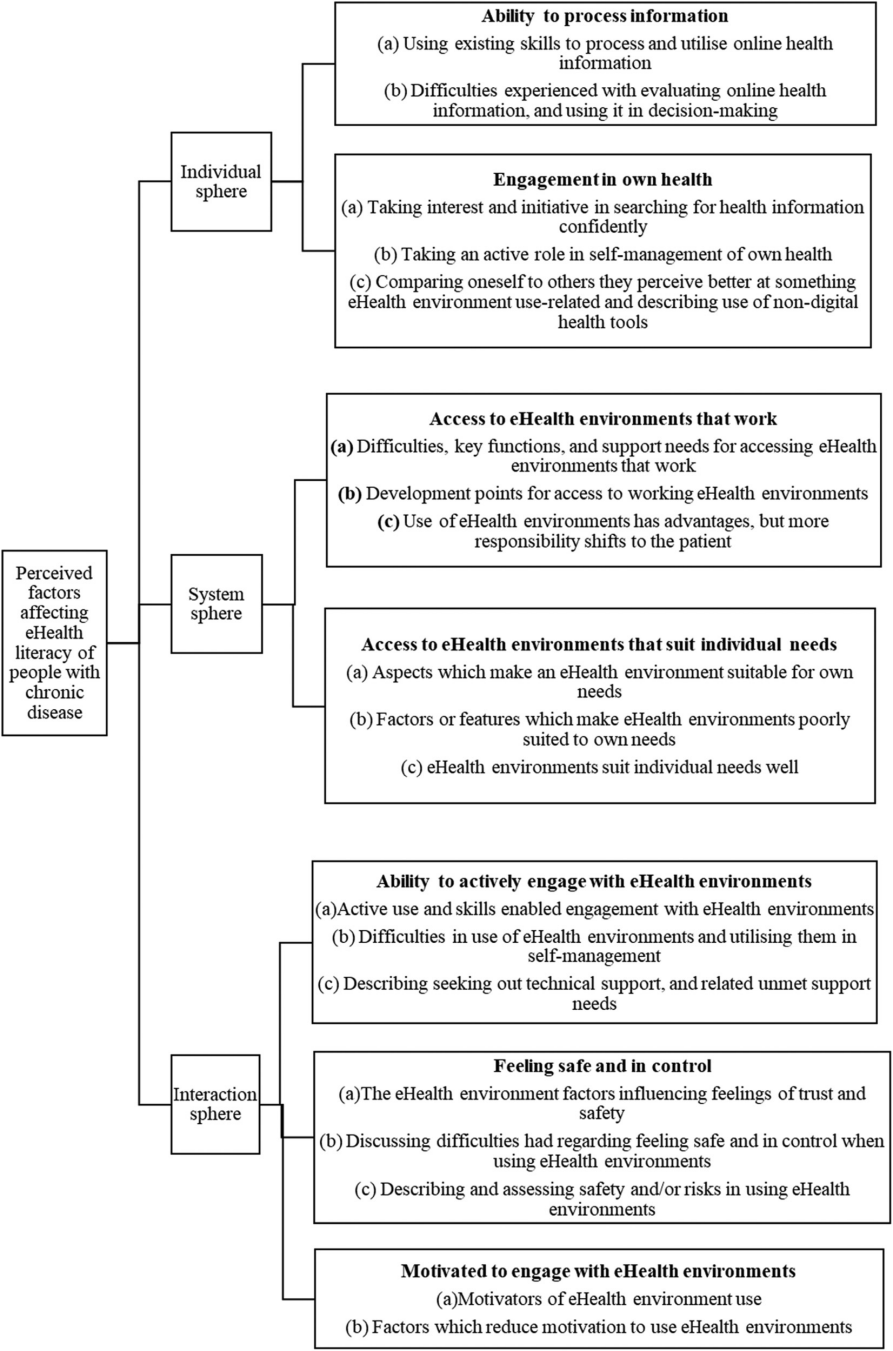
Graphic representation of the main categories generated by each domain of the eHealth Literacy Framework and discussed in writing below.

The results will be displayed below in a graphical form (Figure 1), followed by an elaboration on the ideas presented.

### Individual aspects in eHealth literacy

Individual aspects regarding eHealth literacy comprised of (a) the ability to process health information online and (b) an engagement in the management of one's own health, which seemed to play a crucial role in participants’ ability to process health information online.

The participants described the ability to process information from the perspectives of utilising existing skills to process and apply online health information and difficulties experienced with evaluating online health information and using it in decision-making. The participants had mostly broad IT skills, supporting active engagement with eHealth environments, ranging from basic computer and/or smartphone use skills to advanced technical skills. The advanced skills included, for example, the ability to adapt to different use-logic platforms easily, seek out and apply necessary instructions and data protection materials and work experience in the IT field. Most participants described how they use not only technical skills but also critical thinking, language competencies and logical reasoning skills:

Well, the language issue is one that causes challenges in (determining if a health information source is reliable) and well, yes, the fact that if it is a little unknown subject area – it is impossible to say whether this is now true or fiction. Fiction or fact? If it really is an unknown subject area. (man, 70 years)

The participants showed source criticism, which was also not limited to a critical review of information but extended to an assessment of the trustworthiness and legitimacy of eHealth environments (such as services, tools) and their providers and the potential risks and opportunities associated with them:

I cannot really say what would bring the most uncertainty. When you know where to go (online), it is not that uncertain. In a sense, if one was an insecure type of person, and unaware what is trustworthy and what is not, then the internet would be full of false information. When you know where to go, it is not unsafe (to use eHealth environments in promoting own health). (woman, 38 years)

The participants, however, had difficulties finding and choosing good-quality information sources, keeping up with information regarding the existing eHealth environments and their chronic disease, utilising information provided in an unfamiliar language or about a less familiar topic as well as determining the relevance of the information for their situation especially for those with multiple chronic diseases. Making the distinction between a good or a bad quality – a trustworthy or a non-trustworthy – online source of health information was perceived difficult.

The participants described the engagement in own health from the perspectives of (a) taking interest and initiative in searching for health information confidently, (b) taking an active role in the self-management of their own health and (c) lastly also comparing themselves with others they perceived to have stronger skills. Most participants described instances or tendency to take the initiative in searching for health information and described instances where they confidently used online resources in self-management of their health. The participants also described how they generally play an active role in self-management of their own chronic disease.

#### System-related aspects in eHealth literacy

The system aspects comprised the ability of the individual to access eHealth environments that works in a way that the participant can utilise it and corresponds to their individual needs. The barriers to access identified by the participants and the factors which made eHealth environments unsuitable for their needs were similar.

The participants described the access to health environments that work from the perspectives of (a) barriers and facilitators related to accessing eHealth environments that work, (b) development points for access to working eHealth environments and (c) eHealth environments providing advantages but shifting more responsibility to the patient. Consistent, logical-feeling and reliable technical functionality, conciseness and use of common language were considered key in both accessible and individually suitable eHealth environments. Such environments were utilised in self-management of their chronic disease and general health. Accuracy and transparency of information were also considered important and determined if the participants found the information to suit their needs. The use of eHealth environments, for example, for monitoring and sharing health data was perceived advantageous. However, the participants noted that with digitalisation of healthcare services and health information provision, more responsibility is shifted to the patient. The participants were open-minded, and their active user experiences aided them in making use of different types of eHealth environments:

I guess there is not much else to it, if there are always new (eHealth environments) then I do familiarise myself with them and one should – that creates the confidence in the use, one has to just be brave to do and experience. (man, 70 years)

The participants described their access to eHealth environments that suit their individual needs from the perspectives of (a) aspects that make an eHealth environment suitable for their needs, (b) factors and features which make eHealth environments poorly suited to their needs and (c) elaborated on eHealth environments which suit their individual needs well. There were different types of barriers recognised by the participants in terms of access to eHealth environments that work and correspond to individual needs. There were barriers to access discussed, which caused the eHealth environments to not work well for the participants. Barriers resulting from the environments not being suitable for those with physical impairments were abundant. Such barriers included, for example, inadequate font size or colour use, the environment not being user-friendly or as easily understandable when zooming in:

…and then this one (issue with using eHealth environments) is just the light green (texts or icons). You can't even see it as a poorly sighted person . . . Well, the text needs to be sort of like - like a couple of notches bigger. That already helps a lot. (woman, 69)

Barriers to access not related directly to physical ability included, for example, environment not using common language, not being user-friendly and having unappealing layouts. Factors or features which made some eHealth environments unsuitable to the participants’ needs included poor usability and visual outlook, inaccurate or outdated information and having to re-log the same personal or medical details during every session in some eHealth platforms. Clear instructions in many forms, such as text instructions, video instructions and voice recording instructions, were considered necessary:

It really is about how you are able to use it. Is it easy to use, readable and if it has 10 or 35 pages. So if it gets too long it's a bit like, it needs to be limited – that it doesn’t go on and on so you’d even need to go brew some coffee in between. (woman, 62)

### Aspects related to the interaction between the individual and the system in eHealth literacy

The interaction aspects comprised of (a) the ability of the individual to engage with eHealth environments, (b) the individual feeling safe and in control and (c) motivation to engage with eHealth environments. Both one's physical and mental abilities, as well as personal motivations to engage with eHealth environments, enabled the active use of eHealth environments in promoting one's health and self-managing their health-related needs.

The participants described their ability to actively engage with eHealth environments from the perspectives of (a) their active use and skills enabling engagement with eHealth environments, (b) difficulties they experienced trying to utilise eHealth environments in self-management and (c) describing experiencing technical support needs. The participants possessed the necessary skills to engage with a variety of eHealth environments. All the participants had experiences using multiple different eHealth environments and were actively using them to promote their health and manage their health-related needs. The commonly mentioned types of eHealth environments used included online information sources, multifunctional patient organisation websites, national and local eHealth data and service platforms. Overall, the participants were actively engaging with eHealth environments, but there were multiple instances where technical assistance was needed in using them:

I do have to say the digital sensor in this chronic disease management has revolutionised the whole ordeal, and especially since you can link it with your own appointments, it has made it much easier and – being able to see the graphs and such – well, if there is a repeated issue it's easier to intervene by myself. (woman, 31 years)

The participants described the aspects relating to (a) feeling safe and in control from the perspectives of eHealth environment factors influencing feelings of trust and safety, (b) difficulties they had regarding feeling safe and in control when using eHealth environments and (c) assessing safety and risks in using eHealth environments. The participants explained several factors that influenced the feeling of safety and control when using eHealth environments, such as the eHealth environment type, and the creators of the environment.

Most participants mentioned things they do to feel more secure when online and seemed to adopt quite a high degree of personal responsibility when assessing and acting in eHealth environments. They considered matters like treatment quality, data protection and prevalence of disinformation. The participants also discussed, for example, wanting to seek decision support also from healthcare professionals over only trusting non-person aids or advice provided online, and some had doubts over the quality of eHealth treatment in comparison to in-person treatment:

Depends on the service provider. So generally yes, the mentioned things I find trustworthy, but as said – it depends on the provider. (man, 70 years)

The participants described the motivation to engage with eHealth environments from the perspectives of (a) motivators of use and (b) factors reducing motivation to engage with eHealth environments. The participants’ ability to engage with eHealth environments was at times impaired by experienced technical difficulties or by struggling with unclear or varying user logic of different environments.

Some participants needed technical support on occasion. Additionally, the participants were not always able to apply the eHealth environments in the self-management of their chronic disease. Many described physical ability-related barriers for use, most of which were related to poor sight. The participants were motivated to use eHealth environments when they perceived the use beneficial, and physical ability-related accessibility barriers were found demotivating. The participants felt eHealth environments had many potential benefits, especially in terms of services, for example, saving them time, money and effort in moving around or waiting on the phone:

Of course there is the thing – that they work. So, if I have the (local patient portal) application and I have the application for it – I have it on my tablet and smartphone. And basically, I can be connected to good things through that. In a sense the opportunity to connect (with local healthcare providers) moves with me, because I always have at least my phone on me. (man, 62 years)

Factors which – if realised – would help them utilise eHealth environments more included user-friendliness of the environments as well as online or in-person guidance for using new eHealth platforms and digital medical devices meant to be used in self-management of their health. Overall, most participants experienced the eHealth environments which enabled them to manage their health-related needs in a more flexible manner, but some found the use strenuous:

They do take quite a lot – there are other things to life than being ill – quite a lot of effort. You would have to fill its own (application or platform record) for every diagnosis, really it is the combination when you have multiple illnesses, it is darn arduous. (woman, 50 years)

## Discussion

### Summary of the main findings

This interview study aimed to describe the aspects related to the individual, the system and their interaction, which can affect eHealth literacy from the perspective of people living with one or multiple chronic diseases. Given the skewed nature of the sample, with a female majority, the participants’ well-rounded skill set and active self-management of their disease or diseases, generalisation of the findings to the broader population is neither warranted nor intended. On the individual level, these participants described a variety of skills to be key in using eHealth environments, such as IT skills, critical thinking and ability to evaluate offered content, as well as traits such as curiosity and willingness to try.

Despite their digital competence and proactive self-management, participants encountered challenges when using eHealth environments. Accessibility was deemed a significant system-related aspect to improve. Additionally, the participants described barriers to use, such as lacking digital devices or having limited physical abilities like impaired vision, which resulted in support needs for accessing eHealth environments. Although our participants actively interacted with eHealth systems and utilised them to self-manage their health, using eHealth environments was experienced to increase the patients’ responsibility in the management of their own health. While most of our highly capable sample perceived this positively, some participants felt overburdened.

Our findings raise concerns: even the most competent individuals can experience challenges, casting doubt on the adoption of eHealth environments among more vulnerable patient groups. These insights highlight the importance of prioritising the end-user perspective when designing eHealth environments, with more consideration for cognitive and physical challenges experienced by those with chronic illnesses. The eHealth systems should cater to people with different physical impairments to avoid barriers to use. Furthermore, ensuring broad access to support and assistive devices for navigating these platforms is essential. The workload needed to use the environments, considering the available mental resources of a person with chronic illness, should be individually assessed in the planning of care pathways. The ultimate goal should be to facilitate the management of chronic conditions through eHealth environments, rather than further burdening individuals.

### Comparison with previous research

People with chronic diseases possessing good eHealth literacy skills have been shown to have more positive health outcomes such as being able to better understand their diseases, have better communication with healthcare professionals and self-manage their own health better than those who have poorer eHealth literacy skills,^18^ aligning with findings from this study. The participants in our study were skilled users of eHealth environments, and most participants described digital communication strategies they had used to communicate and share health-related data with their healthcare providers. The participants in this study described good IT skills and did not experience issues with operating a computer and using search functions, unlike in another study where such difficulties were prevalent among participants with chronic disease.^37^

Our study participants showed motivation to engage with many eHealth environments. They considered eHealth environments to be helpful tools. However, it must be noted that the participants were generally active users of such environments and possessed relatively strong IT and critical appraisal skills. In our study, the participants’ education level was quite high, with all participants holding either a basic or higher education degree. Similarly, in a study by Stellefson et al. with participants having a chronic disease, the participants consisted of highly educated people, and a positive association between level of education and eHealth literacy was shown.^38^

The participants also expressed seeking knowledge about their chronic disease and utilising it in their consultations with healthcare professionals. It is also important to note that the participants often reported self-efficacy and confidence in utilising eHealth environments in managing their health-related needs, enabling them to actively engage with eHealth environments. In a previous systematic review about digital interventions for chronic disease management, the people who actively engaged with digital interventions reported greater self-efficacy in self-management of their disease.^15^

Our study participants showed motivation to engage with many eHealth environments. They considered eHealth environments to be helpful tools. However, it must be noted that the participants were generally active users of such environments and possessed relatively strong IT and critical appraisal skills. The participants also expressed seeking knowledge about their chronic disease and utilising it in their consultations with healthcare professionals. The participants in our study did express that the user-friendliness of the eHealth environments could be improved, which would motivate them to utilise them more. For future research, the stratification of user experiences by people living in urban versus rural environments would be interesting, as different levels of eHealth services were recently observed between urban and rural populations in Finland.^39^

Albeit Finns are active users of the internet,^28^ and different types of environments – such as the national patient portal (OmaKanta), the digital health library (Terveyskirjasto), the digital health self-management platform (Health Village) and telemedicine – are made available,^25^ many citizens have shared having experienced issues in using digital health services^40^ A large-scale population-based Finnish study showed that a higher age and poorer digital competence were hindering the use of digital health services. Additionally, digital competence was able to hinder the age-related decline in digital health service use until 80 years of age.^41^ The participants in our study had both good digital competence, were under 80 years of age and can be considered to have less age-related decline in digital service use than the general population.

The study participants did not describe having impactful functional disabilities, except in terms of vision for some. A population-based study has shown that older Finnish adults with less functional disabilities can perceive digital health services more useful than those with more functional disabilities and that good ability to learn supported use.^42^ It was also proposed that when digital health services were made accessible, this could stimulate learning.^42^ Our study participants showed the ability to learn and use eHealth environments, but even this skilled sample brought about the accessibility concerns. Additionally, it should be noted that chronically ill people with lower eHealth literacy have been shown to experience barriers to using eHealth services such as patient portals and should be provided with adequate support especially in the beginning of portal adoption.^18^ While developing eHealth literacy skills is important, the eHealth environment should be tailored to match the eHealth literacy of the intended users, as this impacts their utilisation.^43^

In our study, some system aspects of eHealth environments made them less fitting for our participants and in practice made it harder to engage with those environments. These consisted of physical impairment-related barriers and other types of experienced difficulties. Our finding on the importance of accessibility supports a recent population-based study in Finland, which showed that those with deteriorated vision had significantly lower odds of using digital services than those without vision impairment.^44^ With increasingly aging populations, aging-related decline in physical abilities and IT skill level should be considered in eHealth environment design. The other types of difficulties described by the participants included difficulties with finding suitable eHealth resources and distinguishing their quality, keeping up with online health information and environments as well as making use of information provided in foreign or technical language or about a previously unfamiliar topic. Some of these latter barriers have been seen in a study regarding eHealth environment use in people with rheumatic diseases, that is, difficulty with navigating in online environments and evaluating relevance and accuracy of information.^37^

### Limitations

We acknowledge that there are some limitations in our study. Participants were mainly recruited through electronic channels in collaboration with patient organisations, which may partially explain the participants’ high involvement with eHealth environments, and their high subjective eHealth literacy. It has been recently demonstrated that the self-reported eHealth literacy skills level does not strongly equate to actual demonstrated eHealth literacy.^45^ Even though the purpose was not to make an objective estimation of the participants’ true eHealth literacy level, it must be noted that the participants’ actual competence level may not correspond with their self-reported skills and competence.

It must be considered that our sample was skewed, consisting largely of highly educated individuals, especially given that a higher education level may be positively associated with good eHealth literacy.^38^ To have a more realistic understanding of the aspects affecting eHealth literacy among those living with chronic diseases, future studies should focus on groups with lower eHealth literacy and educational backgrounds. Additionally, some gender bias may have occurred, as most participants were women, so the gender representation was not balanced. This is important to note as it has been observed that in some cases, women use more healthcare services than men.^46^

The interpretation of the representativeness of our sample in relation to chronic conditions and their stability is restricted due to our collection of sensitive health and functioning information being solely based on participants’ voluntary sharing. However, our data covered experiences from individuals with diverse chronic conditions, including some of the most common chronic diseases – although prominently diabetes – as well as those with multimorbidity and a few rare diseases. It is noteworthy that certain prevalent chronic conditions, such as cancer, cardiovascular diseases and memory disorders, were not discussed, potentially limiting a comprehensive depiction of the experiences related to eHealth environments among individuals with common chronic diseases.^20^

Due to the qualitative study design, the findings of this study cannot be easily generalised to a wider population. Generally, a more thorough methodological triangulation would have been ideal. For this study, a power calculation was not used, which is normally related to qualitative interviews in which the data saturation of the interviews has an important role in determining the sample size,^47^ as also in our study. The intended in-depth reflections of the subject matter were obtained from the participants. The use of the eHealth Literacy Framework may have introduced some confirmation bias, albeit working in an existing paradigm was perceived useful, as grounding the analysis in some existing theory helps contextualise and compare the findings. The data was coded by one coder, albeit the coding was actively discussed within the research team throughout the data coding period. The interviews being conducted by one interviewer pose a possibility of interviewer bias, albeit measures described above were taken to reduce such bias.

## Conclusions

This study shows that even people who have a high subjective eHealth literacy experience and actively use eHealth environments face many types of challenges and barriers in using eHealth environments to manage their health-related needs. The participants utilised a wide set of skills and characteristics to manage and overcome such barriers, but as eHealth solutions become more widely utilised, more consideration must be given to those whose skills or other physical abilities are more limited. Regarding this, organisations offering eHealth information, tools and services have the opportunity and ethical mandate to tailor their online environments to be accessible. More research is needed to obtain a richer understanding and better catered eHealth environments for those living with chronic disease. Cooperation and joint initiatives are needed to understand the role of eHealth literacy in peoples’ ability to utilise the eHealth solutions offered. As treatment and other types of costs (such as productivity losses) of chronic diseases amount to a significant portion of the healthcare costs, promotion of self-management with eHealth solutions is of key importance.

## Supplemental Material

sj-docx-1-dhj-10.1177_20552076231216395 - Supplemental material for Perceptions of Finns with chronic diseases about factors affecting their eHealth literacy: A qualitative interview study Click here for additional data file. Supplemental material, sj-docx-1-dhj-10.1177_20552076231216395 for Perceptions of Finns with chronic diseases about factors affecting their eHealth literacy: A qualitative interview study by Alisa Tamminen, Lotta Virtanen, Timo Clemens, Janna Nadav, Petra Saukkonen, Emma Kainiemi, Tarja Heponiemi and Anu-Marja Kaihlanen in DIGITAL HEALTH
