# Supplementary material for: Perceptions of Finns with chronic diseases about factors affecting their eHealth literacy: A qualitative interview study
Source: Digit Health. 2023 Nov 27;9:20552076231216395. doi: 10.1177/20552076231216395 (PMC10683406; doi:10.1177/20552076231216395)
Supplement: sj-docx-1-dhj-10.1177_20552076231216395 - Supplemental material for Perceptions of Finns with chronic diseases about factors affecting their eHealth literacy: A qualitative interview study [file sj-docx-1-dhj-10.1177_20552076231216395.docx]

**Supplementary materials**

Alongside this manuscript, there are two appendices to be published as supplementary materials. Appendix A, titled as ‘Appendix A: Coding tables with the main categories of code, arranged per domain of the eHealth Literacy Framework.’, contains the coding tables with the main categories of code, with three tables titled as ‘Table 1: Individual sphere code main categories’, ‘Table 2: System sphere code main categories’, ‘Table 3: Interaction sphere code main categories’. Appendix B contains the interview guide in English, titled as ‘Appendix B: Interview guide in English’.

**Appendix A:** Coding tables with the main categories of code, arranged per domain of the eHealth Literacy Framework.

Table 1: Individual sphere code main categories

| **Ability to process information** | **N** | **Engagement in own health** | **n** |
| --- | --- | --- | --- |
| Using existing skills to process and utilise online health information | 8 | Taking interest and initiative in searching for health information confidently | 28 |
| Difficulties experienced with evaluating online health information, and using it in decision-making | 57 | Taking an active role in self-management of own health | 15 |
|  |  | Comparing oneself to others they perceive better at something eHealth environment use-related and describing use of non-digital health tools | 6 |

Table 2: System sphere code main categories

| **Access to eHealth environments that work** | **n** | **Access to eHealth environments that suit individual needs** | **n** |
| --- | --- | --- | --- |
| Difficulties, key functions, and support needs for accessing eHealth environments that work | 42 | Aspects which make an eHealth environment suitable for own needs | 25 |
| Development points for access to working eHealth environments | 18 | Factors or features which make eHealth environments poorly suited to own needs | 25 |
| Use of eHealth environments has advantages, but more responsibility shifts to the patient | 16 | eHealth environments suit individual needs well | 15 |

Table 3: Interaction sphere code main categories

| **(3) Ability to actively engage with eHealth environments** | **N** | **(4) Feeling safe and in control** | **n** | **(5) Motivated to engage with eHealth environments** | **n** |
| --- | --- | --- | --- | --- | --- |
| Active use and skills enabled engagement with eHealth environments | 87 | The eHealth environment factors influencing feelings of trust and safety | 72 | Motivators of eHealth environment use | 71 |
| Difficulties in use of eHealth environments and utilising them in self-management | 36 | Discussing difficulties had regarding feeling safe and in control when using eHealth environments | 66 | Factors which reduce motivation to use eHealth environments | 41 |
| Describing seeking out technical support, and related unmet support needs | 34 | Describing and assessing safety and/or risks in using eHealth environments | 54 |  |  |

**Appendix B:** Interview guide in English

**Interview guide**

*Before the start of the interview It was repeated that the participants could refuse to answer an of the questions, and sharing only what they feel comfortable with.*

**Section I: Background questions**

1. Age:

2. Gender:

3. Nationality:

4. Highest completed level of education (i.e. middle school, high school or vocational education, higher education such as technical university or research university):

**Section II: Questions regarding eHealth environments**

*The following questions concern digital health literacy, i.e. the skills needed to use the various health-related information sources, tools and services offered via the internet. Sources of information offered via the internet are i.e. the patient organization's internet pages, the Terveykylä service, health-related blogs or magazines and health encyclopaedias read via the internet such as Duodecim. Tools offered via the internet are i.e. electronic medical records and health and exercise smartphone applications. Health services offered via the Internet include, for example, the OmaKanta service, remote consultation with a healthcare professional and the electronic renewal of prescriptions. In this interview, these health-related information sources, tools and services are discussed together as "eHealth environments".*

**eHealth environments: awareness, use and perceived benefits**

- Have you used eHealth environments before (services, information sources, tools)? If yes, then what? If not, why not?
- What benefits have you experienced using eHealth environments; what motivates use? You can also mention examples.
- In addition to these, are there other eHealth environments that you are aware of or have heard of?
- Would you describe - what kind of eHealth environment is useful for you?

🡪 If you need to specify: What characteristics do you feel are important in eHealth environments?

**Ability to function in eHealth environments**

- How do you search for information related to your health via the internet (e.g. applications, websites)?

🡪 If not searching on the internet: If you need to get information related to your health, how do you proceed with the matter (e.g. do you look for information in books, do you contact your health care provider, something else?)

- What skills do you have that enable you to utilise eHealth environments to promote your health (i.e. self-management of disease, general health information)?
- What kind of challenges do you experience in using eHealth environments?
- What kind of support would you like to find and use eHealth environments that are useful for you? What could make you utilise electronic health environments more?

**Assessing quality**

- How do you evaluate the quality of eHealth environments (source of information, service, etc.)?
- What characteristics do you consider to be the hallmarks of a good quality eHealth environment?
- What kind of characteristics do you consider to be the hallmarks of a low-quality eHealth environment?
- What kind of challenges do you experience when evaluating the quality of an eHealth environment (that is, when you think about whether the source or service is of good or poor quality)?

**Trust in eHealth environments**

- Do you consider health information obtained via the internet to be reliable? How about healthcare services used via the internet (such as remote consultation with a doctor, electronic renewal of prescriptions, etc.)?
- What evokes trust in an eHealth environment (e.g. website, service, application)?
- Does the use of eHealth environments feel safe?

**Confidence in using eHealth environments**

- What causes (especially/most) uncertainty in the use of eHealth environments?
- What could increase your confidence in using electronic health environments?

**Free speech**

Is there anything else you have in mind – regarding the use of eHealth environments as part of promoting your health – that I have not yet asked about, but you would like to share your thoughts on?

*At the end of the interview it was asked if the participants wanted to comment on a summary of the interview, and if they wanted to hear about the interview results.*
